# Transcriptome profiling and analysis of patients with esophageal squamous cell carcinoma from Kazakhstan

**DOI:** 10.3389/fgene.2024.1249751

**Published:** 2024-03-18

**Authors:** Aigul Sharip, Saule Rakhimova, Askhat Molkenov, Ainur Ashenova, Ulan Kozhamkulov, Ilyas Akhmetollayev, Andrei Zinovyev, Yuri Zhukov, Marat Omarov, Mukhtar Tuleutaev, Venera Rakhmetova, Joseph D. Terwilliger, Joseph H. Lee, Zhaxybay Zhumadilov, Ainur Akilzhanova, Ulykbek Kairov

**Affiliations:** ^1^ Center for Life Sciences, National Laboratory Astana, Nazarbayev University, Astana, Kazakhstan; ^2^ National Center for Biotechnology, Astana, Kazakhstan; ^3^ Institut Curie, PSL Research University, Paris, France; ^4^ Multidisciplinary Medical Center, Astana, Kazakhstan; ^5^ Department of Internal Diseases, Astana Medical University, Astana, Kazakhstan; ^6^ Sergiеvsky Center, Columbia University, New York, NY, United States; ^7^ Division of Medical Genetics, New York State Psychiatric Institute, New York, NY, United States; ^8^ Department of Psychiatry and Department of Genetics and Development, Columbia University, New York, NY, United States; ^9^ Departments of Epidemiology and Neurology, Columbia University, New York, NY, United States; ^10^ School of Medicine, Nazarbayev University, Astana, Kazakhstan

**Keywords:** esophageal squamous cell carcinoma, RNA-seq, next-generation sequencing, transcriptomics, analysis of differentially expressed genes, bioinformatics, Kazakhstan

## Abstract

Esophageal squamous cell carcinoma (ESCC) is the predominant subtype of esophageal cancer in Central Asia, often diagnosed at advanced stages. Understanding population-specific patterns of ESCC is crucial for tailored treatments. This study aimed to unravel ESCC’s genetic basis in Kazakhstani patients and identify potential biomarkers for early diagnosis and targeted therapies. ESCC patients from Kazakhstan were studied. We analyzed histological subtypes and conducted in-depth transcriptome sequencing. Differential gene expression analysis was performed, and significantly dysregulated pathways were identified using KEGG pathway analysis (*p*-value < 0.05). Protein-protein interaction networks were constructed to elucidate key modules and their functions. Among Kazakhstani patients, ESCC with moderate dysplasia was the most prevalent subtype. We identified 42 significantly upregulated and two significantly downregulated KEGG pathways, highlighting molecular mechanisms driving ESCC pathogenesis. Immune-related pathways, such as viral protein interaction with cytokines, rheumatoid arthritis, and oxidative phosphorylation, were elevated, suggesting immune system involvement. Conversely, downregulated pathways were associated with extracellular matrix degradation, crucial in cancer invasion and metastasis. Protein-protein interaction network analysis revealed four distinct modules with specific functions, implicating pathways in esophageal cancer development. High-throughput transcriptome sequencing elucidated critical molecular pathways underlying esophageal carcinogenesis in Kazakhstani patients. Insights into dysregulated pathways offer potential for early diagnosis and precision treatment strategies for ESCC. Understanding population-specific patterns is essential for personalized approaches to ESCC management.

## 1 Introduction

Esophageal cancer (EC) globally ranks as the sixth leading cause of cancer mortality, and it significantly contributes to disease burden with its high mortality rate due to invasive manifestation and poor survival prognosis (Lu et al., 2012; Tungekar et al., 2018; Liu WJ. et al., 2023). Despite significant burden on cancer epidemiology, EC remains one of the least studied cancer types. Esophageal cancer consists of two histologic types: esophageal squamous cell carcinoma (ESCC), which lines the surface of the esophagus, and esophageal adenocarcinoma (EAC), which mainly occurs in the cells of mucus-secreting glands in the esophagus (Zhang et al., 2015; Doghish et al., 2023). Its prevalence is notably pronounced in developing areas, accounting for nearly 80% of all instances (Fan et al., 2020; Liu C-Q. et al., 2023, Jemal et al., 2010). Specifically, in the defined “esophageal cancer belt” which stretches from the Middle East to Northeast China, almost 90% of all EC cases are classified as ESCC (Zhang, 2013). In Kazakhstan, esophageal cancer ranks sixth in prevalence among all cancer types, and ESCC is the predominant subtype (Igissinov et al., 2012). In this study, we restrict our discussion to ESCC. The majority of ESCC patients are diagnosed in the advanced metastatic stage at their first screening because there is a limited number of screening tests for early diagnosis (Jemal et al., 2010). Once diagnosed, the five-year survival rate ranges from 10% to 25% (Fan et al., 2020; Fernandes et al., 2006; Liu C-Q. et al., 2023; Zhang et al., 2015). The occurrence and fatality rates of esophageal cancer remain significant, with 5.90 new diagnoses and 5.48 deaths per 100,000 individuals across the globe in 2017 (Fan et al., 2020). The causes of esophageal cancer vary depending upon geographical locations, and some regions are witnessing a continual escalation in both its incidence and mortality. Despite notable advancements in diagnostic and therapeutic strategies, the survival rates for patients with advanced ESCC have not shown significant improvement (Liu C-Q. et al., 2023).

Over the last decade, the advent of high-throughput genomic and proteomic analyses led to the discovery of a few potential driver mutations in ESCC (Tungekar et al., 2018) have uncovered a small number of potential driver mutations in ESCC (Lin et al., 2014; Song et al., 2014; Zhang et al., 2015; Cui et al., 2017). Yet, the genetic and molecular alterations contributing to ESCC development are still not well-characterized, underscoring the need for a comprehensive pathological exploration to develop more effective diagnostic and therapeutic strategies. Therefore, a comprehensive investigation of the pathological mechanisms would give new insights into more effective diagnosis and treatment options (Zhang et al., 2015). With recent advancement of next-generation sequencing technologies, the RNA-seq approach has become a powerful tool for comprehensive characterization of the entire transcriptome of tissues. In this study, we conducted whole RNA sequencing of tumor tissues and differentially expressed gene screening with functional enrichment analysis of transcriptomic profiles in Kazakhstani patients to investigate the distinct gene expression patterns of ESCC. The findings will contribute significantly to the development of early diagnostic biomarkers and personalized therapeutic approaches in ESCC management.

## 2 Materials and methods

### 2.1 Samples and clinical data

All ESCC patients recruited to the study were from the Multidisciplinary Medical Center in Astana, Kazakhstan. Samples were gathered from the Department of Oncological Surgery. Study protocols were approved by the institutional ethics review board of the National Laboratory Astana (protocol #13, 12 March 2014 and protocol #20, 22 September 2017). Informed consent was obtained from all study participants. Tumor tissues were collected from each patient who underwent Ivor-Lewis esophagectomy between 2013 and 2017. None of these patients were treated with chemotherapy or radiotherapy before surgery. After the surgical procedure, tissue samples were immediately frozen in liquid nitrogen and stored at −80°С. Hematoxylin/eosin staining of tissue samples was performed to validate the diagnosis and to determine the pathological grade, metastasis, and cellular content of tumor samples. All tumor samples were more than 80% free of necrosis. Tumor samples were classified based on the tumor-node-metastasis (TNM) classification of the International Union against Cancer, 7th edition (Sobin et al., 2010). Evaluation of tumor differentiation was performed according to histological criteria of the guidelines of the World Health Organization (WHO) Pathological Classification of Tumors (Bosman et al., 2010). Diagnoses of all ESCC cases were histologically confirmed. The following three criteria were used to select patients for this study: confirmed ESCC status, informed consent for research, and Ivor-Lewis esophagectomy. Tumor sample tissues obtained from 22 ESCC patients were subjected to total RNA extraction and sequencing.

### 2.2 RNA preparation and sequencing

Total RNA was extracted from approximately 60 mg of tissue for each of the 22 tumor samples using the RNAiso Plus (Takara) and purified using DNase I kit (QIAGEN) based on manufacturer instructions. Quantification of the RNA yields was performed by NanoDrop ND1000 (Thermo Fisher Scientific, Waltham, MA). The quality of the RNA was evaluated by the Agilent 2,100 Bioanalyzer (Agilent, Santa Clara, CA). A cDNA library was prepared according to the protocol of TruSeq RNA Sample Preparation based on manufacturer instructions. The cDNA library of each sample was assessed and validated on Qubit (Qubit ds DNA HS assay kit) and on an Agilent Bioanalyzer 2,100 (HS DNA kit). Further, the cDNA library was normalized and after pooling was then hybridized on a flow-cell v3 (TruSeq E Cluster Kit version 3). Finally, RNA paired-end sequencing was performed on an Illumina HiSeq2000 platform according to the standard protocol using TruSeq SBS Kit (TruSeq SBS Kit version 3—HS). A PhIX control library was used as an in-spike for each line (Huang et al., 2011). Transcriptome sequencing data are available publicly at the NCBI Sequence Read Archive (http://www.ncbi.nlm.nih.gov/sra/) under accession number PRJNA608223.

### 2.3 Acquisition of transcriptomic data for normal esophageal tissue

Total RNA-seq data from healthy esophageal squamous epithelial tissue were downloaded from the GEO database (https://www.ncbi.nlm.nih.gov/geo/). Gene expression profiles of 11 healthy esophageal squamous epithelial tissues were extracted from the atlas of RNA sequencing profiles for normal human tissues (Suntsova et al., 2019). The sequencing and total RNA extraction protocol for the selected sample can be found at the GEO database under accession number GSE120795 (https://www.ncbi.nlm.nih.gov/geo/query/acc.cgi?acc=GSE120795). Paired-ended reads were downloaded from SRA Run Selector at NCBI under accession number PRJNA494560 on 30 March 2020. Raw RNA-seq data in FASTQ format were aligned to a reference genome (Homo_sapiens.GRCh38.94.gtf) using STAR tools (version 2.1.3) (Dobin et al., 2013).

### 2.4 Immune deconvolution of ESCC and normal EC samples

We estimated the relative abundance of specific cell types from bulk tissue transcriptomic profiles using CIBERSORTx algorithm, which is a machine learning computational framework for the assessment of cellular abundance and cell type-specific gene expression from bulk tissue gene expression profiles (Chen et al., 2018; Newman et al., 2019).

As input, CIBERSORTx requires a “signature matrix” comprised of barcode genes are enriched cell type of interest. In this study, we used LM22 signature matrix consisting of 547 genes that estimates the fraction of 22 immune cell types, including mainly T cells, B cells, neutrophils, macrophages, natural killer cells and myeloid subsets (Chen et al., 2018). The gene expression profiles of tumor ESCC and normal EC samples were uploaded to the CIBERSORTx website (https://cibersortx.stanford.edu/) and data analysis (Job type: “Impute cell fractions”) was run in default settings, in absolute mode using the LM22 signature over 1,000 permutations with quantitative normalization disabled (Braun et al., 2020; Zhao et al., 2020). To evaluate the deconvolution confidence, CIBERSORTx calculated several quality control metrics, including deconvolution *p*-value and Pearson correlation coefficient (Chen et al., 2018).

### 2.5 The identification of differentially expressed genes

A modified Tuxedo Suite protocol was used to identify differentially expressed transcripts between tumor and normal conditions (Pertea et al., 2016). All alignments and mapping were performed using the STAR tool (Dobin et al., 2013). Sequenced reads were aligned to human reference genome from the ENSEMBL database (Homo_sapiens.GRCh38.94.gtf) for gene expression analysis. The quantification and transcript assembly of RNA-seq alignments were accomplished using StringTie (Pertea et al., 2016). Raw RNA-seq data were normalized using the *DeSeq2* package in R (version 1.24.0, https://bioconductor.org/packages/release/bioc/html/DESeq2.html) for differential expression analysis (Love et al., 2014). The DEGs between ESCC samples and normal esophageal tissue were identified using *DeSeq2* package. The raw *p*-values of genes were adjusted with the Benjamin and Hochberg (BH) method. The genes with absolute value of log-fold changes of gene counts (|log2FC|≥1) and adjusted *p*-value <0.05 were selected as significant DEGs, and these thresholds were accepted as the cut-off values for statistical significance. Moreover, the cross-section of the DEGs according to tumor stage was calculated and the results were visualized as a Venn diagram using an online tool (http://bioinformatics.psb.ugent.be/webtools/Venn/).

### 2.6 Reverse transcription for cDNA preparation and RT-PCR

Real-time RT-PCR was used to validate gene expression level of selected DEGs genes, which has significant downregulation and upregulation. Total RNA was extracted from approximately 60 mg of tissue using the RNAiso Plus (Takara) and purified using DNase I kit (QIAGEN) based on the instructions of manufacturer. The quantification of the RNA yields was performed by NanoDrop ND1000 (Thermo Fisher Scientific, Waltham, MA). The quality of the RNA was evaluated by the Agilent 2,100 Bioanalyzer (Agilent, Santa Clara, CA). To obtain complementary DNA (cDNA) on an RNA template, a reverse transcription reaction was performed using the TaqMan™ Reverse Transcription Reagents kit (Applied Biosystems, United States) with random hexanucleotide primers in accordance with the manufacturer’s instructions. RT-PCR was performed under the following thermal cycling conditions: 10 min at 25°C, 60 min at 37°C, 5 min at 95°C. The concentration of the obtained cDNA samples was determined by the spectrophotometric method using the NanoDrop device. RT-PCR was performed in a volume of 20 µL containing 10 µL Master Mix, 4 µL Primer Mix, 1 µL Taq polymerase, 1 µL cDNA, and 4 µL MilliQ water. Each target gene’s expression level was standardized relative to the expression of glyceraldehyde-3-phosphate dehydrogenase (GAPDH), which served as the internal reference gene. The two-step amplification program included 1 cycle at 95°С, 3 min for preliminary denaturation; 40 cycles of the first amplification step at 95°С, 10 s and the second annealing-extension step for 40 s at a temperature of 60°С. Real-time RT-PCR cycling was performed on Bio-Rad CFX-96 system (Bio-Rad Laboratories, Hercules, CA, United States). The fluorogenic signal emitted was collected during the anneal extension step. Three replicates of the assay were performed to assess reproducibility and the coefficient of variation were statically calculated. The comparative method of measuring threshold cycle ΔCt, which is calculated based on the difference between the Ct values, was used to calculate the relative level of gene expression in the samples compared to the control. Two downregulated genes (TNXB and MUC5B) and one upregulated gene (MAGEA4) have been selected for RT-PCR validation experiment. The following Taqman gene expression assays (FAM-MGB labelled) were obtained from Thermo Fisher Scientific: MAGEA4 (Hs00751150_s1), TNXB (Hs00372889_g1), MUC5B (Hs00861595_m1). The selection of genes for validation was determined by considering multiple criteria. Our primary criterion was the gene expression level, focusing on the genes with the highest upregulation and the most significant downregulation, determined by their log-fold changes in gene counts (log2FC), while ensuring their adjusted *p*-value was <0.05. We refrained from choosing non-coding RNA for several reasons, including their limited functional characterizations, intricate regulatory mechanisms, and the technical limitations associated with their analysis. The level of gene expression of following genes in 3 ESCC samples were assessed using quantitative real-time reverse transcriptase PCR (RT-qPCR) using TaqMan technology.

### 2.7 Functionаl annotation and pathway-enrichment analysis

Functional analysis of DEGs was performed using the Database for Annotation, Visualization and Integrated Discovery (DAVID) online tool version 6.8 (Huang et al., 2009a; b) to assign significant DEGs to their associated biological annotation with Gene Ontology (GO; www.geneontology.org) (Ashburner et al., 2000; The Gene Ontology, 2017). Pathway analysis was performed using the Kyoto Encyclopedia of Genes and Genomes (Ogata et al., 1999; Kanehisa and Goto, 2000) and the Reactome pathway database (Fabregat et al., 2018). Default parameter settings were used for the DAVID tool. Significantly enriched pathways were defined by *p*-values <0.05 calculated based on hypergeometric distribution with the BH correction. All significantly enriched terms were visualized in bubble charts using the *ggplot2* package version 3.1.0 in R (Villanueva and Chen, 2019). The richness factor was computed as a ratio between the number of enriched gene number and the number of background genes of the same term. Heatmaps for DEGs were created for unsupervised clustering using *pheatmap* package version 1.0.12 in R (Kolde, 2019). KEGG pathway analysis of DEGs was performed using *GAGE* package in R (Luo et al., 2009; Luo and Brouwer, 2013), and a whole set of DEGs was selected for functional enrichment analysis.

### 2.8 Prоtein-prоtein interactions (PPI) network cоnstruction

The most significant 500 DEGs were screened for protein-protein interactions (PPI) using the Search Tool for the Retrieval of Interacting Genes/Proteins database version 11.5, and a combined score >0.4 was used as the criterion to establish the PPI network (Szklarczyk et al., 2015). The data of the PPI network were exported from STRING and imported into Cytoscape version 3.9.1 software for visualization (Shannon et al., 2003). Each protein in the network served as a node, and the degree and betweenness centrality were calculated using the CentiScape version 2.2 plug-in Cytoscape (Scardoni et al., 2009; Scardoni et al., 2014; Wang et al., 2019; Yang et al., 2019).

Highly interconnected regions or clusters (modules) were determined using the MCODE plugin (version 2.0.2) (Bader and Hogue, 2003) in the PPI network. The degree cut-off and k-score were set to 2. Identified clusters with a score >10 was used to create a sub-network. The Cytoscape plug-ins ClueGO (version 2.5.9) and CluePedia (version 1.5.9), which enable GO and pathway enrichment analysis in a network (Bindea et al., 2009; Bindea et al., 2013), were applied to conduct functional enrichment analysis and visualization. The results from the GO and KEGG databases were combined, and in the process, the same color was used to represent similar functional terms (Wang et al., 2019).

## 3 Results

### 3.1 Clinical characterization of samples and tissues

From 2013 to 2017, a total of 184 patients with Esophegeal cancer (EC) were hospitalized in the Multidisciplinary Medical Center, Astana, Kazakhstan. Surgical resection was performed on 54 patients and 3 patients refused surgical treatment. Of the EC patient cohort, 22 ESCC patients with full and high-quality RNA-seq data were selected for further transcriptomic profiling of ESCC tissue. Of these patients, 13 were men and the mean ± SD age was 65.73 ± 8.26 years. Most (82.0%) of these patients were Kazakhs. Most (86.4%) were diagnosed with advanced stages (III-IV stage; 77% were in stage III) and 74.5% had dysphagia levels III to IV. Cancer was localized predominantly to the middle and lower thirds of the thoracic part of the esophagus. The clinical features of the 22 patients are shown in Table 1, and further details are presented in Supplementary Table S1. A histopathological study of the ESCC patients indicated that the following three histological types were observed: moderate dysplasia with invasive growth, high-grade dysplasia, and cancer pearls. According to the histological type of ESCC, moderately differentiated squamous cell carcinoma with infiltrative growth and with keratinization and high-grade dysplasia were prevalent.

**TABLE 1:** Clinical features of the 22 Kazakhstani esophageal cancer patients.

Characteristic	Statistics
Age, years, mean ± SD (range)	65.72 ± 8.26 (48–74)
Gender	
Male	13 (59%)
Female	9 (41%)
Nationality/Ethnicity	
Kazakh (Asian)	18 (82%)
Russian (Caucasian)	4 (18%)
TNM classification^a^	
I/II/III/IV	1/2/17/2
N0/N1/ND	6/4/12
M0/M1	22/0

^a^ More information on TNM classification and description of each parameter can be found in the work of Sobin and his colleagues (Sobin et al., 2010).

### 3.2 Evaluation of immune deconvolution of tumor and normal EC samples

We determined the relative abundance of 22 immune cells for each samples using CIBERSORTx (Chen et al., 2018; Newman et al., 2019). The comparative summary of immune cell fractions is shown in Supplementary Figure S1. We observed that immune cells, such as T cells CD4 memory resting and macrophages M2 were enriched across all samples. It was revealed that memory B cells, CD8 T cells, regulatory T cells and activated dendritic cells were differentially enriched in different samples. Our analysis demonstrated that there is significant difference in correlation coefficient between normal EC samples (in average 0.05) and tumor ESCC samples (in average 0.27) that was determined by Pearson correlation analysis. There is some positive association between signature score and immune cell infiltration in ESCC samples.

### 3.3 Assessment of immune cell composition in normal and tumor EC samples

To evaluate the immune landscape of esophageal cancer (EC), we analyzed the relative proportions of 22 immune cell types in each sample using CIBERSORTx (Chen et al., 2018; Newman et al., 2019). The resulting immune cell fraction for each sample is represented in Supplementary Figure S1.

Throughout all samples, substantial enrichment of resting memory CD4 T cells and M2 macrophages was observed. However, the enrichment of memory B cells, CD8 T cells, regulatory T cells, and activated dendritic cells varied noticeably among different samples, highlighting heterogeneity in immune responses. Our subsequent analysis revealed a significant difference in the correlation coefficient for immune cell infiltration between normal EC and tumor ESCC samples. Normal EC samples exhibited an average correlation coefficient of 0.05, while tumor ESCC samples had a greater mean coefficient of 0.27, as determined by Pearson correlation analysis. This suggests a positive association between the signature score and degree of immune cell infiltration, particularly in ESCC samples.

### 3.4 Identification of differentially expressed genes

Based on RNA-seq of 22 ESCC tissue samples, a total of 6,689 significant DEGs with 4,633 upregulated genes and 2056 downregulated genes were identified. The list of significant DEGs for tumor tissue versus normal esophageal tissue is shown in Supplementary Table S2. When we compared ESCC tissues against normal esophageal tissues by tumor stage, we observed a total of 3,243, 4,464, 6,756, and 1,904 DEGs for tumor stage I, II, III, and IV stages versus the expression in normal esophageal tissues, respectively (Table 2).

**TABLE 2:** The number of identified differentially expressed genes (DEGs) between ESCC samples and normal esophageal tissue based on tumor stages.

	All ESCC samples	I	II	III	IV
Number of samples for each stage		1	2	17	2
Number of upregulated DEGs	4,633	1,332	2,594	4,636	1,114
Number of downregulated DEGs	2056	1911	1870	2,120	790
Total DEGs	6,689	3,243	4,464	6,756	1904

The number of overlapping and unique DEGs for each tumor stage is shown in Supplementary Figure S2. We further examined 1,002 genes that were consistently aberrantly expressed (Supplementary Figure S2A); these comprised 505 downregulated and 491 upregulated genes (see Supplementary Figures S2B, C).

### 3.5 Quantitative real-time RT-PCR validation

We conducted quantitative real-time RT-PCR (RT-PCR) to validate the results obtained from RNA-Seq analysis in three ESCC samples by assessing the mRNA expression of these genes. Among the significantly expressed DEGs listed in Supplementary Table S2, we chose one upregulated gene (MAGEA4) and two downregulated genes (TNXB and MUC5B) for validation. The relative gene expression of these selected genes was assessed using the comparative Ct method and normalized to GAPDH. Comparing the expression of these genes with reference genes, we observed that TNXB and MUC5B were downregulated, while MAGEA4 was upregulated across all three samples (as shown in Supplementary Figure S3). Additionally, all selected genes exhibited similar expression levels across the three ESCC samples. The concordance between RT-PCR and RNA-seq results confirmed that the differential expression of TNXB, MUC5B and MAGEA4 and that the findings from RNA-seq analysis were creditable.

### 3.6 Functional gene enrichment analysis of DEGs in ESCC

To determine the relevant pathways altered in ESCC pathogenesis, functional enrichment analysis was carried out using the DAVID tool for identification of associated Gene Ontology terms and KEGG/Reactome pathways. Given the small number of samples evaluated in the present study, statistical analyses at a single-gene level may lack power, we thus performed pathway analysis to try to glean some biological interpretation using clusters of the genes that are differentially expressed. Enriched KEGG pathways of DEGs yielded 42 upregulated and two downregulated KEGG pathways for DEGs of ESCC samples versus normal esophageal tissue. The list of these pathways with involved genes for each pathway is shown in Supplementary Table S3. The upregulated pathways included the oxidative phosphorylation, rheumatoid arthritis, neurogenerative diseases, viral protein interaction with cytokine and cytokine receptor, coronavirus disease and others. The most significantly downregulated pathways were tight junction and adherens junction; these are associated with degradation of the extracellular matrix.

**FIGURE 1:**
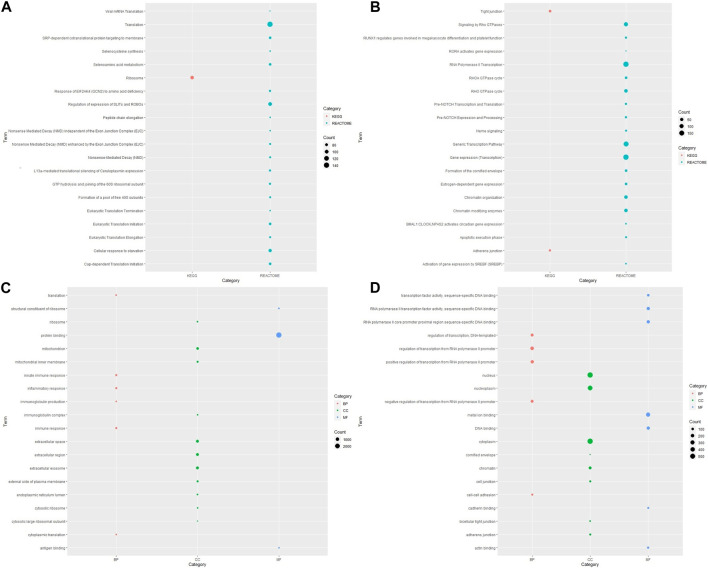
Visualization of pathway and Gene Ontology enrichment analysis for DEGs from ESCC samples compared with normal esophageal tissue. KEGG and Reactome pathways for **(A)** upregulated and **(B)** downregulated genes; GO-enriched terms for **(C)** upregulated and **(D)** downregulated genes. The y-axis reflects the significantly enriched pathways/GO terms, and the x-axis reflects functional groups (KEGG/REACTOME/GO). The size of the bubble is associated with the number of assigned genes to a pathway/GO term. A larger bubble size is linked to a larger number of genes of the specific term. KEGG, Kyoto Encyclopedia of Genes and Genomes; GO, Gene Ontology; BP, biological process; CC, cellular component; MF, molecular function.

The analysis of pathway functional enrichment revealed a total of 20 significantly enriched pathways (Figures 1A, B) and Gene Ontology (GO) terms (Figures 1C, D) for the DEGs between ESCC and normal esophageal samples.

**FIGURE 2:**
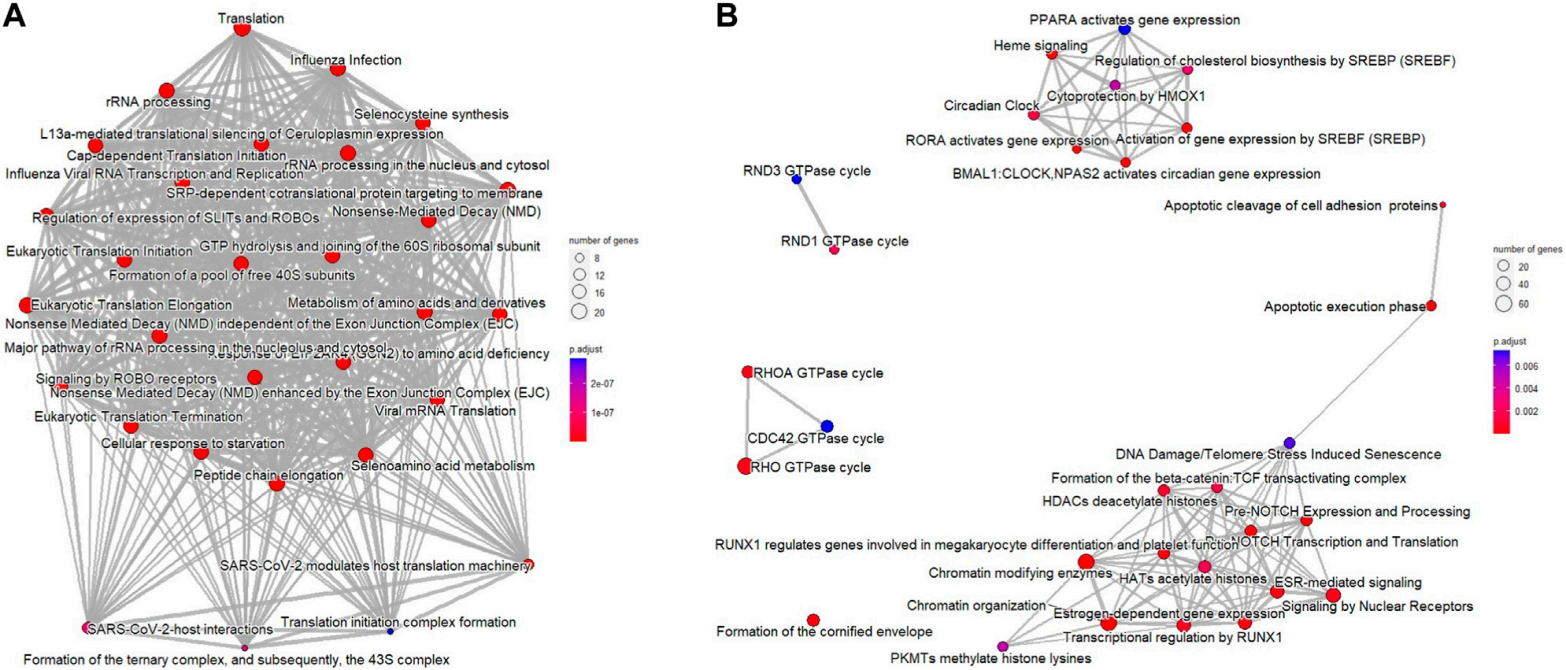
Reactome pathways for DEGs from ESCC samples compared with normal esophageal tissue using the ReactomePA package. **(A)** upregulated DEGs; **(B)** downregulated DEGs. The bubble color represents the adjusted *p*-value for specific Reactome terms, and the size of the bubble is associated with the number of assigned genes to specific Reactome terms.

In addition, functional enrichment analysis of Reactome pathways was performed using ReactomePA package (Yu and He, 2016). Figure 2 shows that the number of selected genes associated with the specific Reactome pathway is larger than expected, and the additional Reactome pathways analysis identified DNA repair, histone function, WNT and NOTCH signaling pathways, and apoptotic execution phase as enriched in downregulated DEGs of ESCC samples (Figures 2A, B).

### 3.7 Protein-protein interaction (PPI) network analysis

**FIGURE 3:**
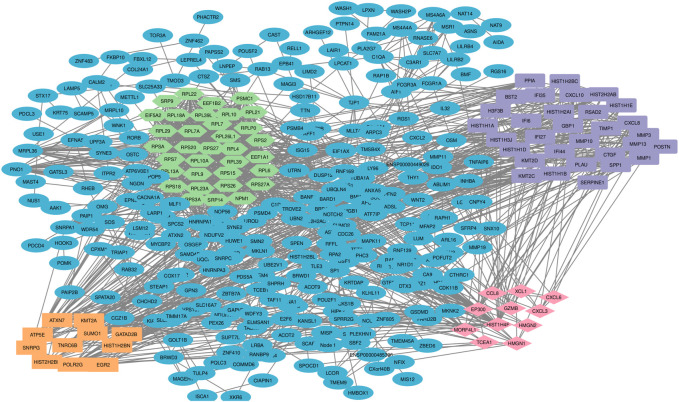
Protein-protein interaction network and sub-network analysis for DEGs in ESCC. The pivotal DEGs were mapped onto a protein-protein interaction network, revealing four distinct modules of closely connected nodes. Each module had specific functions and are involved in esophageal carcinogenesis. Using the STRING dataset, 446 DEG-encoded proteins were mapped to a PPI network. Topology analysis was conducted using the MCODE plug-in and four significant sub-networks (modules) were determined. Light green color shows the DEG-encoded proteins of module 1, purple color shows the DEG-encoded proteins of module 2, orange color shows DEG-encoded proteins of module 3 and red purple color shows DEG-encoded proteins of module 4. PPI: protein-protein interaction; DEGs: differentially expressed genes.

**FIGURE 4:**
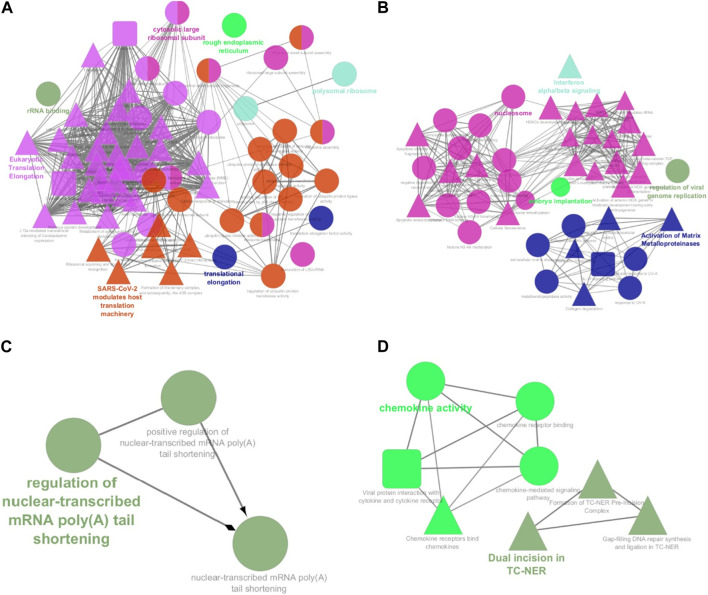
Functional annotation of the four significant modules from the PPI network analysis. The functional enrichment analysis of the four sub-networks was carried out using the ClueGo and CluePedia plug-ins. **(A)** Module 1 consists of 34 proteins that are principally linked to translational elongation and ribosomal functions; **(B)** Module 2 consists of 31 proteins that are linked to histone function, activation of matrix metalloproteinases and interferon signalling; **(C)** Module 3 consists of 11 proteins that are linked to mRNA regulation. **(D)** Module 4 consists of 11 proteins that are linked to chemokine activity. Circles represent Gene Ontology terms, round rectangles represent enriched KEGG pathways, and triangles represent enriched REACTOME pathways. PPI: protein-protein interaction.

Among the 500 genes that were differentially expressed between tumor samples and normal esophageal tissue, a total of 446 genes (nodes) were mapped to the PPI network with 1907 edges. The central nodes were chosen with a threshold criterion of degree >10 within the top betweenness centrality nodes to help identify the shortest paths. We have identified top 20 hub genes based on their high degree of connectivity within the network using CytoHubba plugin with Degree method (Supplementary Table S4). The highest-ranked genes are usually considered as hub genes in the network, suggesting they likely have key roles in the functions of the network. These genes, we believe, could consequently offer valuable targets for early detection and therapeutic interventions for ESCC. Four modules, closely connected nodes, were identified using the MCODE plug-in in Cytoscape and colored with different colors (Figure 3). Module 1 (light green) consisted of 34 nodes and 526 edges; module 2 (purple) consisted of 31 nodes and 128 edges; module 3 (orange) consisted of 11 nodes and 17 edges; and module 4 (red purple) consisted of 11 nodes and 17 edges. Supplementary Table S4 provides an organized list of the genes identified within key modules of the protein-protein interaction network. The hub genes, highlighted via their intensive network connections, are thought to play potentially significant roles in the network functions. Functional enrichment analysis of these four modules revealed that module 1 was significantly associated with translational elongation and ribosomal functions (Figure 4A). Module 2 was associated with histone function, activation of matrix metalloproteinases and interferon signalling (Figure 4B). Module 3 was principally associated with mRNA regulation (Figure 4C). Module 4 was mostly associated with chemokine activity (Figure 4D).

**FIGURE 5:**
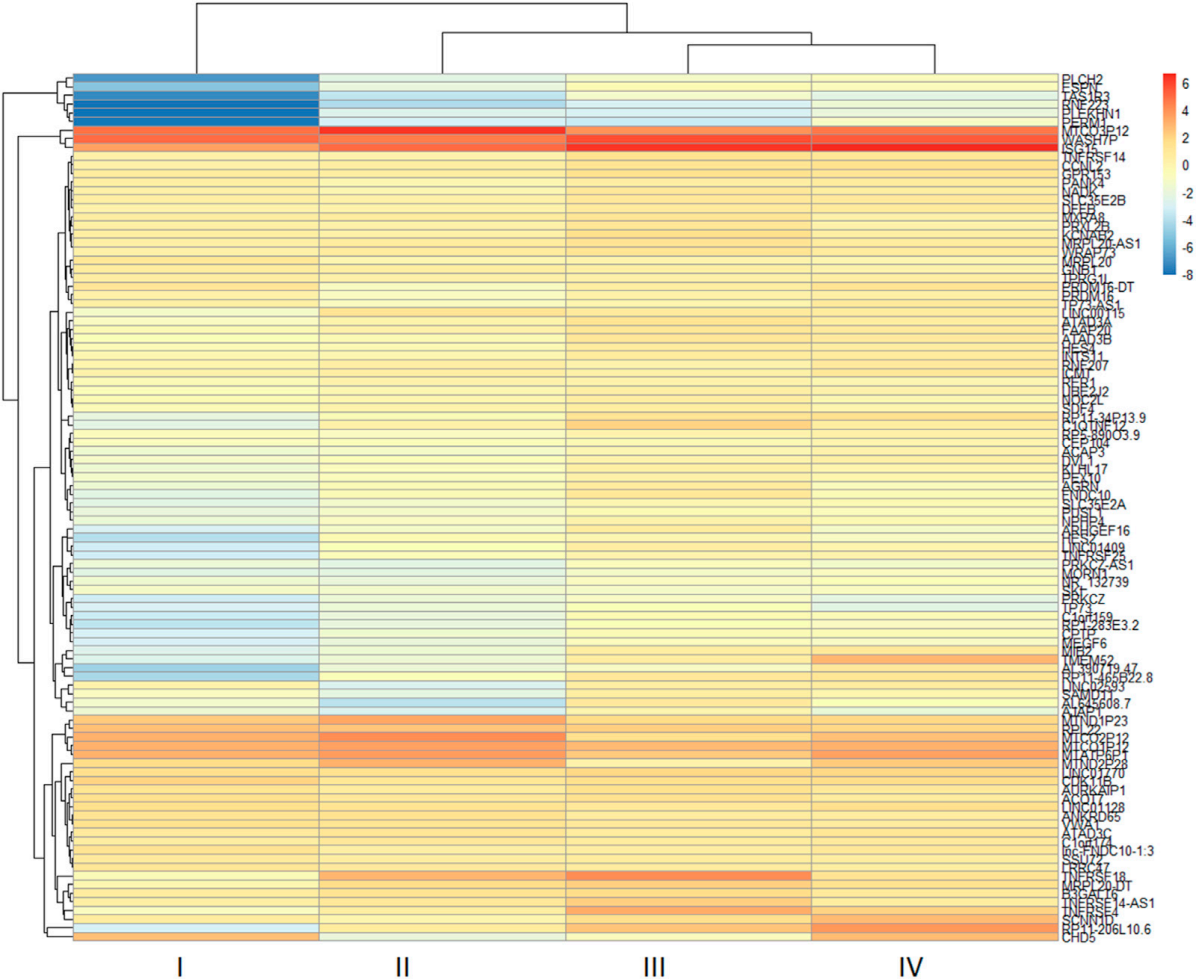
Expression heatmap of the top 100 DEGs in different tumor stages (I-IV) from ESCC tissues. Hierarchical clustering of the pivotal DEGs by variance in ESCC stages groups the samples based on gene signatures according to tumor stages of samples. The top 100 DEGs were determined in ESCC samples compared with healthy esophageal tissue and these genes were used to construct a heatmap using the *pheatmap* package in R based on rowVar function. The dendrogram at the top represents the sample clustering. The rows indicate the genes, and the columns indicate the tumor stages. Red and blue colors represent upregulated and downregulated genes, respectively. DEGs: differentially expressed genes.

**FIGURE 6:**
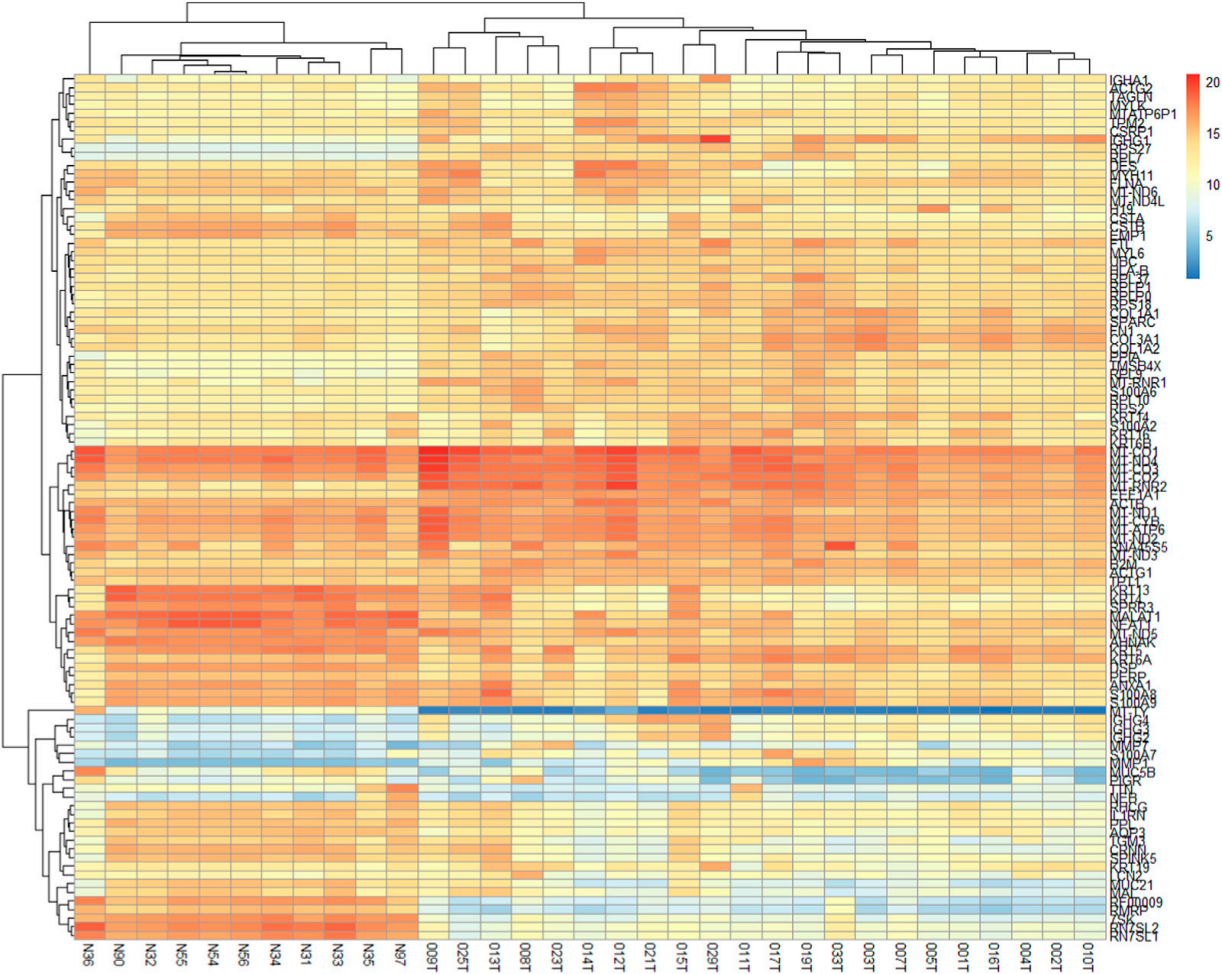
Expression heatmap of the 100 DEGs across 22 ESCC samples and 11 normal EC samples. These 100 genes provide clear clusterization between tumor ESCC samples and healthy esophageal tissue samples. Heatmap was constructed using the *pheatmap* package in R based on rowVar function. The dendrogram at the top represents the sample clustering. The rows indicate the genes, and the columns indicate the samples. Red and blue colors represent upregulated and downregulated genes, respectively. DEGs: differentially expressed genes.

The two-dimensional hierarchical clustering analysis for the top 100 DEGs based on variance at different stages of ESCC shows that these gene signatures cluster the samples according to tumor stages (Figure 5; Supplementary Table S6). Clustering analysis across tumor ESCC samples and healthy 11 samples demonstrate clear clusterization of two groups (Figure 6; Supplementary Table S7).

**FIGURE 7:**
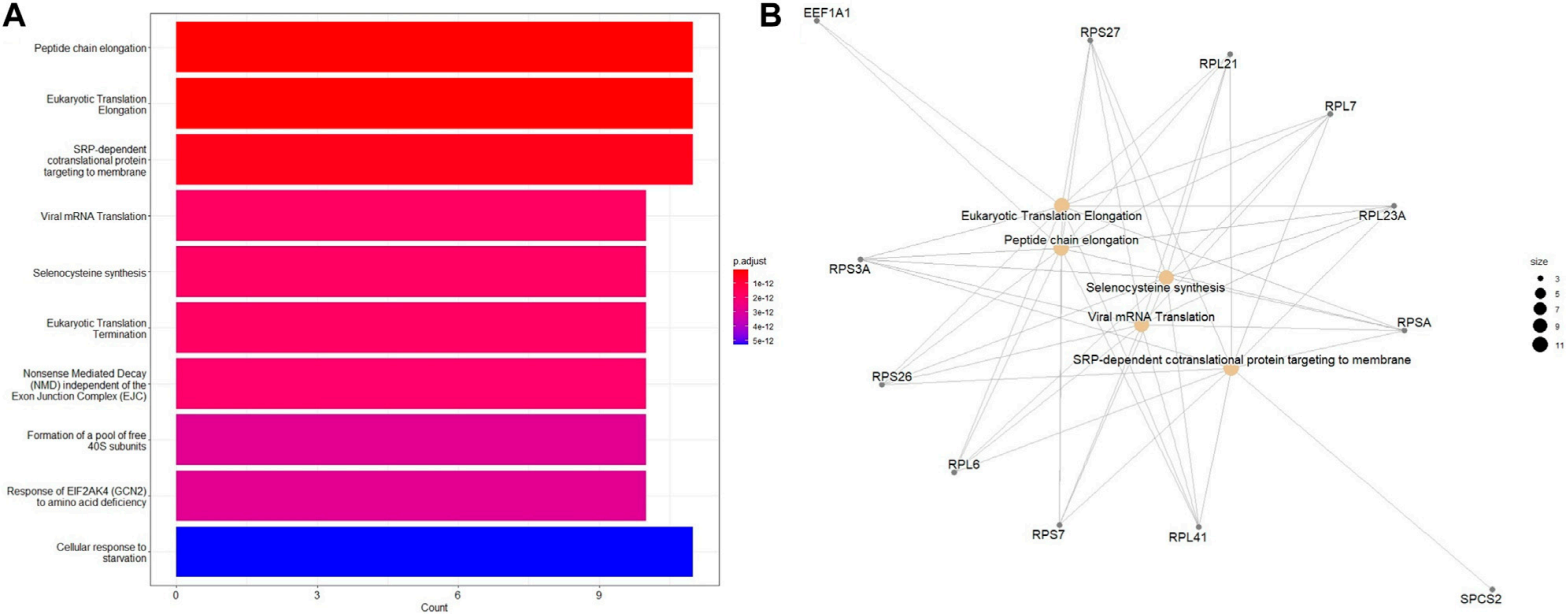
Enriched Reactome pathways of the 100 top DEGs in different tumor stages of ESCC tissues using the ReactomePA package. **(A)** Barplot and **(B)** Emapplot. The bubble color is associated with adjusted *p*-value for specific Reactome terms and the size of the bubble is linked to the number of assigned genes to specific Reactome terms. A larger bubble size reflects a larger number of genes of the specific term.

Additional functional enrichment analysis based on ReactomePA package revealed that, in addition to DNA repair identified from the earlier analysis, eukaryotic translation elongation and peptide chain elongation, viral mRNA translation were enriched in downregulated DEGs of ESCC samples (Figures 7A, B). A subsequent functional enrichment analysis for the top 100 DEGs revealed that these genes play a central role in transcription regulation, DNA repair, DNA replication, and chromosome stability. Moreover, pathways for degradation of the extracellular matrix (ECM), collagen degradation, and tight function were significantly enriched, indicating the importance of ECM in cancer pathogenesis.

## 4 Discussion

Recent investigations employing whole-genome sequencing and whole-exome sequencing have revealed mutations in multiple genes, including TP53, CDKN2A, FAT1, NOTCH1, PIK3CA, KMT2D, and NFE2L2, previously linked to esophageal squamous cell carcinoma (ESCC) (Sasaki et al., 2016). Despite this recognition of genes or proteins that are linked with the development of ESCC, a comprehensive understanding of the pathogenic processes involved remains elusive. The existing gap in our knowledge of the molecular and cellular underpinnings of ESCC and the lack of potential target genes creates a pressing need for more rigorous investigation into the molecular mechanisms that initiate and propagate ESCC, which will undoubtedly improve diagnosis and therapeutics of ESCC. Considering the notable variance in ESCC occurrence across diverse geographical populations (Tungekar et al., 2018), it becomes crucial to gain population-specific insights into ESCC’s molecular mechanisms for unveiling promising therapeutic targets. In response to this need, in our study, we conducted transcriptome profiling and screening for differentially expressed genes, paired with a functional enrichment analysis applied to 22 esophageal cancer tissue samples sourced from Kazakhstan patients to investigate the distinct gene expression patterns of ESCC.

In our endeavor to elucidate the genetic basis of ESCC in Kazakhstani patients and discover potential biomarkers for early detection and targeted treatment strategies, we initially identified differentially expressed genes (DEGs) in ESCC, this was subsequently followed by an extensive functional enrichment analysis on these significant DEGs. Insights gained from this analysis demonstrated 42 upregulated and two downregulated KEGG pathways within the ESCC samples (*p*-value <0.05; Supplementary Table S3). The majority of these upregulated pathways related to innate and adaptive immune responses, including pathways of oxidative phosphorylation, rheumatoid arthritis, neurogenerative diseases, viral protein interaction with cytokine and cytokine receptor, and coronavirus disease. This underscores the central role of immune pathways in ESCC manifestation (Nicolau-Neto et al., 2018; Huang and Fu, 2019). In contrast, two downregulated pathways in the ESCC tissues derived from Kazakhstani patients were found to be the adherens and tight junctions, which are associated with the degradation of the extracellular matrix (ECM). The ECM, a network of diverse macromolecules, plays a significant role in key cellular processes such as proliferation, migration, differentiation, and apoptosis. The ECM is strongly regulated during embryonic development, while it often exhibits dysregulation in disease states, including cancer. Pathologies in the ECM are known to contribute to cancer progression via several mechanisms. For one, dysregulated ECM can stimulate angiogenesis and inflammation, encouraging the formation of a tumorous microenvironment (Lu et al., 2011). Furthermore, abnormal ECM can modulate cancer pathogenesis by influencing the behavior of stromal cells, such as immune cells, endothelial cells, and fibroblasts (Lu et al., 2011; Lu et al., 2012). Additionally, abnormal ECM can promote cell transformation and metastasis. The transformative actions of the ECM are largely mediated by metalloproteinases, a vital enzyme needed for ECM remodeling (Lu et al., 2012). Interestingly, an increased activation of matrix metalloproteinases was observed in the top 100 DEGs (Supplementary Table S7) within the study, which suggests a role in facilitating tissue invasion, growth factor production and tumor angiogenesis (Walker et al., 2018).

We employed protein-protein interaction network (PPI) analysis for aiding in understanding the complex landscape of interactions among our identified significant differentially-expressed genes (DEGs). Our analysis focuses the 500 most significant DEGs, based on adjusted *p*-values. Leveraging the STRING database and Cytoscape plug-ins, these genes were subsequently classified into four distinct modules. Consequent functional enrichment analysis of these four modules unveiled connections to pathways related to esophageal cancer progression, multiple research studies corroborate with these findings. Specifically, Module 1 has shown significant connections to translational elongation and ribosomal functions (Figure 4A; Temaj et al., 2022), Module 2 is linked to histone function, activation of matrix metalloproteinases, and interferon signaling (Figure 4B; Jiao et al., 2014; Mittal et al., 2016; Ozkan and Bakar-Ates, 2020), Module 3 is primarily associated with mRNA regulation (Figure 4C; Teng et al., 2022), and Module 4 is chiefly related to chemokine activity (Figure 4D; Nicolau-Neto et al., 2018). Each of these modules seems to have a unique role in the progression of esophageal cancer.

Given the role of innate and adaptive immunity in ESCC development having been adequately discussed, our focus here shifts towards understanding the role of histone modifications in cancer progression. The regulation of gene expression is tightly controlled by histone acetylation and deacetylation, processes governed by histone acetyltransferases and histone deacetylases (HDACs). It should be noted that HDAC inhibitors have illustrated anticancer properties (Bojang and Ramos, 2014), with high HDAC2 expression correlating with increased aggression in esophageal cancer (Langer et al., 2010). Moreover, histone isoforms are central to carcinogenic processes, with their levels being aberrantly altered in several malignancies, including esophageal cancer (Singh et al., 2018). In particular, Hist1H2AC has been found to be overexpressed in several malignancies whilst being under-expressed in others, including esophageal adenocarcinoma (Kavak et al., 2010). Hence, these studies underscore the role of histone cluster levels in influencing cellular proliferation and tumorigenesis.

In our continuous endeavor to delineate the genetic basis of ESCC in Kazakhstani patients and discover potential early biomarkers for diagnosis and targeted treatment, we conducted additional functional enrichment analysis on the pivotal 100 differentially expressed genes (DEGs). Interesting findings emerged which highlighted the overrepresentation of WNT and NOTCH pathways, both of which are known for their significant roles in cancer progression (Moghbeli et al., 2016; Abbaszadegan et al., 2018). Notably, among the DEGs that exhibited downregulation in our ESCC samples, there was an association with pathways related to the execution phase of apoptosis, suggesting a plausible mechanism for cancer cells to evade apoptosis. Additionally, we observed a decrease in DNA repair pathways and functions related to histone modification in ESCC samples, these findings were supported by relevant research studies (Liu et al., 2022).

Our two-dimensional hierarchical clustering of the top 100 DEGs reveals that several genes are differentially expressed significantly by tumor stages (Figure 5). This suggests that these genes may function as potential biomarkers for the early detection of ESCC. In particular, genes such as MTND1P23, PLCH2, RNF223, PERM1, TAS1R3, and specific TMEM genes exhibit varied levels of expression across different tumour stages, thereby presenting a potential to discern early-stage ESCC from its later stages.

In our research, we employed cutting-edge next-generation sequencing technology to examine the transcriptomes of tumors from 22 Kazakhstani patients with ESCC. While this research offers crucial insights into ESCC, it is worth noting its limitation—the relatively small sample size of patients. For a more comprehensive understanding, future studies could consider further exploring the prognostic implications of MTND1P23, PLCH2, RNF223, PERM1, TAS1R3, and TMEM genes. We presented the exploration of genetic variations and differentially expressed genes in the multi-stage carcinogenesis of ESCC. Several signaling pathways were enriched in our ESCC case series of different stages (I–IV). These findings are useful for comprehensive understanding of the carcinogenesis of esophageal cancer and for designing promising biomarkers for early diagnosis of ESCC.

## Data Availability

The datasets presented in this study can be found in online repositories. The names of the repository/repositories and accession number(s) can be found in the article/Supplementary Material.
